# Pharmaceutical treatment status of patients with COPD in the community based on medical Internet of Things: a real-world study

**DOI:** 10.1038/s41533-024-00371-0

**Published:** 2024-05-10

**Authors:** Peng Wu, Yi-qun Jiang, Feng-li Si, Huan-ying Wang, Xiao-bo Song, Chun-feng Sheng, Xun Xu, Fan Li, Jing Zhang

**Affiliations:** 1 Department of Respiratory and Critical Care, Songjiang Hospital Affiliated to Shanghai Jiao Tong University School of Medicine, Shanghai, 201600, China；Songjiang District Central Hospital, Shanghai, 201600 China; 2 Department of General Medicine, Shanghai General Hospital, Shanghai Jiao Tong University School of Medicine, Shanghai, China; 3 Department of Pulmonary and Critical Care Medicine, Zhongshan Hospital, Shanghai Medical College, Fudan University, Shanghai, People’s Republic of China

**Keywords:** Chronic obstructive pulmonary disease, Drug regulation, Health care economics

## Abstract

This study aimed to investigate the real-world standardisation and adherence of medical treatment regimens in patients with chronic obstructive pulmonary disease (COPD) in the community for making future management strategy. The follow-up data and treatment information of patients with COPD, which were collected through the Management Information Center of COPD (MICCOPD) in 21 community health service centres in Songjiang District, a countryside region of Shanghai. Concordance between the pharmaceutical treatment plan and recommendation of 2017 Global Initiative for Chronic Obstructive Lung Disease (GOLD) report during the follow-up management period, as well as the medication adherence by patients,were analysed. Out of the 2044 patients diagnosed with COPD, 814 patients (39.8%) who had an initial record of medication use were found to meet the inclusion criteria. The most common medication regimens were long-acting beta-agonist plus inhaled corticosteroids (35.9%) and oral bronchodilators (41.9%). Among these 814 patients, 45.7%, 38.0%, 31.6% and 14.6% adhered to the treatment after 6, 12, 18 and 24 months of follow-up, respectively. The concordance rate with the regimens recommended by the 2017 GOLD guidelines was 35.5% at baseline, 35.5% at 6 months, 32.7% at 12 months, 35.4% at 18 months and 37% at 24 months. The compliance and guideline consistency rates of patients with COPD in the community under the management of general practitioners need to be improved. Enhancing general practitioner proficiency in the prevention and management of COPD and increasing patient awareness of the condition, are crucial standardising and improving adherence to initial and follow-up COPD treatments.

## Introduction

Chronic obstructive pulmonary disease (COPD) is a global public health concern and a leading cause of morbidity and mortality due to chronic disease^1^. COPD is characterised by persistent respiratory symptoms and airflow limitation and is a common preventable and treatable condition. The prevalence of COPD increased by 44.2% between 1990 and 2015, and 3.2 million people died from COPD worldwide in 2015, reflecting an increase of 11.6% compared to 1990 and eight times the number of deaths due to asthma^2^. The World Health Organisation predicts that COPD will become the third leading cause of death worldwide by 2030, affecting more than 250 million people and exacting a heavy economic burden. To improve COPD diagnosis and treatment and reduce its prevalence and mortality, the Global Initiative for Chronic Obstructive Lung Disease (GOLD) introduced new personalised treatment protocols in 2017. Despite the existence of international and national guidelines, under- and over-medication continue to persist in clinical practice. The level of compliance with GOLD recommendations also varies across institutions and countries. A cross-sectional study in the USA showed that more than 60% of patients with COPD were not prescribed any COPD-related maintenance medications^3^. Another study confirmed that 32.9% and 58.9% of patients in GOLD groups of A/B and C/D, respectively, were receiving the same treatment regimen recommended by the GOLD guidelines^4^. In contrast, Palli et al. found that some patients with mild COPD were commonly using inhaled medications, including inhaled corticosteroids (ICS)^5^. The results of a questionnaire-based survey conducted by general practitioners (GPs) and pulmonologists suggested that 53% of patients with COPD were not treated according to the 2017 GOLD guideline recommendations^6^. In Spain, more than one-third of patients with COPD have not been prescribed treatments according to the 2017 GOLD guideline recommendations^7^. These studies highlight the frequent inconsistencies between real-world prescription patterns and international guidelines. Poor drug treatment compliance is very common among patients with COPD who receive long-term treatments. Treatment compliance of patients with COPD varies from 29% to 56% across countries^8–10^. The administration of nonstandard treatment and poor compliance of patients with stable COPD are often the main reasons for the deterioration of COPD. Adverse outcomes of nonstandard COPD treatment approaches have been demonstrated in several cohort studies, in which an increased frequency of acute exacerbations was positively associated with an exponential increase in COPD-related costs^7,11^. This finding also underscores the importance of patients with COPD at risk for frequent exacerbations who would benefit from simultaneous and appropriate disease management.

In China, the overall prevalence of COPD is 13.7% among the general Chinese population aged 40 years or older^12^. Chen^13^ reported that 10.8% of patients with COPD were highly compliant with drug treatment during the one-year follow-up management. A majority of the research data on the management of COPD in the stable phase in China published in previous studies was obtained from tertiary or secondary general hospitals^14,15^, and only a few studies reported the long-term management of patients with COPD in the community. Therefore, through the Management Information Centre of Chronic Obstructive Pulmonary Disease (MICCOPD) in Songjiang District, Shanghai, we established an alliance between respiratory specialties and community GPs to promote the prevention and treatment of COPD. Based on the MICCOPD, we analysed the real drug treatment situation of patients with COPD in the community, including drug treatment compliance and consistency with guidelines, to help in optimising the regional management of COPD and improving the prognosis.

## Results

### Patient characteristics and general follow-up survey

**Fig. 1:**
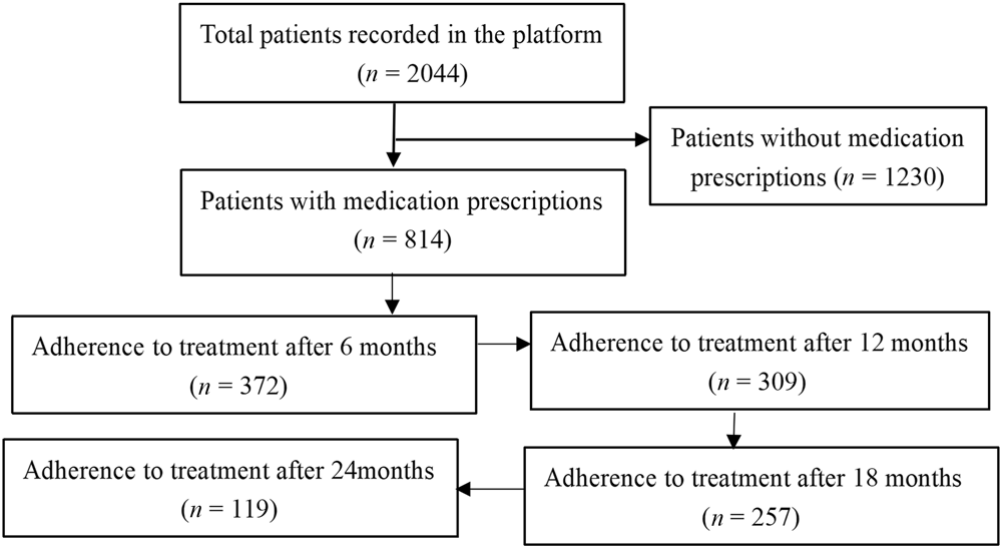
Study flow chart.

Out of the 2044 patients diagnosed with COPD, 814 patients (39.8%) who had an initial record of medication use met the inclusion criteria of these, 372, 309, 257 and 119 patients had adhered to medications after 6 months, 12 months, 18 months and 24 months, respectively. The patients who demonstrate adherence to drug treatment exhibited a declining trend annually (Fig. 1).

In the cohort of 814 patients with initial medication prescriptions, 66.6% were male, 51.1% were aged >75 years, and 87.2% had CAT scores of ≥10. Additionally, 9.2%, 44.0%, 1.6% and 45.2% of the patients were in GOLD groups A, B, C and D, respectively. The most common medication regimens in the overall cohort were LABAs plus ICS (35.9%) and oral bronchodilators (41.9%) (Table 1).

**Table 1:** Baseline characteristics of patients on medication regimens.

Total cases	Patients with medication regimen (*n* = 814)
Sex	
Male (%)	542 (66.6)
Female (%)	272 (33.4)
Age, years (%)	
<65	77 (9.5)
65–75	321 (39.4)
>75	416 (51.1)
Number of smokers(%)	371 (45.6)
CAT score (%)	
<10	104 (12.8)
≥10	710 (87.2)
mMRC grade (%)	
0–1	432 (53.1)
≥2	382 (46.9)
FEV1%pred	53.26 ± 24.98
FEV1/FVC%	51.07 ± 14.09
GOLD grade	
1	69 (8.5)
2	388 (47.7)
3	281 (34.5)
4	76 (9.3)
Number of hospitalisations (%)	
<1	445 (54.7)
≥1	369 (45.3)
Integrated assessment subgroup (%)	
A	75 (9.2)
B	358 (44.0)
C	13 (1.6)
D	368 (45.2)
Medication regimens	
SABA/SAMA	41 (5.0)
LABA/LAMA	51 (6.3)
LABA + LAMA	12 (1.5)
LABA + ICS	292 (35.9)
LABA + LAMA + ICS	13 (1.6)
Oral bronchodilators^a^	341 (41.9)
Others^b^	64 (7.9)

^a^Theophylline sustained-release tablets, compound methoxylamine, etc.

^b^Phlegm-resolving herbs, etc.

*CAT* chronic obstructive pulmonary disease assessment test, *mMRC* modified Medical Research Council, *FEV1* forced expiratory volume in 1 second, *FVC* forced vital capacity, *LABA* long-acting beta-agonist, *LAMA* long-acting muscarinic antagonist, *ICS* inhaled corticosteroids.

### Compliance of initial dosing regimens with guideline recommendations

The overall medication use was consistent with the 2017 guideline recommendations in 289 cases (35.5%). Under- and over-medication were present in 400 (49.1%) and 125 (15.4%) cases, respectively.

**Fig. 2:**
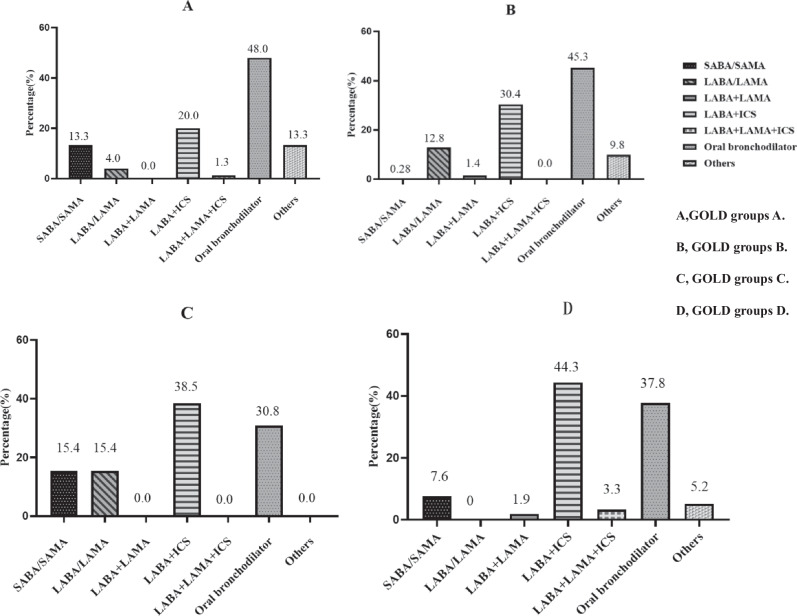
Distribution of specific medication regimens for all patients. **A** GOLD group A. **B** GOLD group B. **C** GOLD group C. **D** GOLD group D.

Analysis of the medication regimens specifically assigned to our study patients revealed that oral bronchodilators were administered to 48.0% and 45.3% of the patients in GOLD groups A and B, respectively, whereas LABAs plus ICS were administered to 20.0% and 30.4% of the patients in GOLD groups A and B, respectively. Only 13.3% of the patients in GOLD group A used SABAs/SAMAs. Only 12.8% of the patients in GOLD group B received LABAs/LAMAs. Oral bronchodilators were administered in 30.8% and 37.8% of the patients in GOLD groups C and D, respectively, with LABAs plus ICS accounting for 38.5% and 44.3% of these patients, respectively. In GOLD group D, 3.3% of the patients received LABAs in combination with LAMAs and ICS (Fig. 2).

The mMRC grades were considerably higher in patients who underwent initial drug therapy in accordance with the guidelines than the scores in patients who were undertreated or overtreated (*P* < 0.001). The overtreated group was younger and had lower CAT scores and fewer hospitalizations in the past year than the other two groups (*P* < 0.05). In addition, FEV1% and FEV1/FVC were higher in the overtreated group than the undertreated and guideline-consistent groups (*P* < 0.05). Undertreated patients were predominantly found in groups B and D, whereas overtreated patients were primarily clustered in groups A and B. Overtreated patients exhibited mild clinical symptoms and lung function (Table 2).

**Table 2:** Baseline characteristics of patients who were consistent with the recommended medication plan and those who were under and over-medicated.

	I	II	III	*P* value
Total cases	*N* = 289	*N* = 400	*N* = 125	
Sex				0.588
Male (%)	199 (68.9)	262 (65.5)	81 (64.8)	
Female (%)	90 (31.1)	138 (34.5)	44 (35.2)	
Age	75.6 ± 8.0	75.9 ± 8.8	73.0 ± 7.8^*#^	0.02
CAT score	16.89 ± 7.34	16.22 ± 5.37	14.75 ± 6.67^*#^	0.03
mMRC grade	1.71 ± 0.88	1.46 ± 0.88^*^	1.29 ± 0.83^*^	<0.001
FEV1%	50.13 ± 21.52	53.39 ± 18.48	60.11 ± 42.99^*^	0.013
FEV1/FVC%	48.96 ± 16.17	51.81 ± 12.96	53.62 ± 11.66^*^	0.023
Number of hospitalisations (%)				<0.001
<1	105 (36.3)	215 (53.8)^*^	125 (100)^*#^	
≥1	184 (63.7)	185 (46.3)	0 (0)	
Integrated assessment subgroup (%)				<0.001
A	49 (17.0)	10 (2.5)^*^	16 (12.8)^#^	
B	51 (17.6)	198 (49.5)^*^	109 (87.2)^*#^	
C	7 (2.4)	6 (1.5)	0 (0)	
D	182 (63.0)	186 (46.5)^*^	0 (0)^*#^	

I, Medication prescription consistent with the 2017 GOLD guidelines; II, under-medication; III, over-medication; *CAT* chronic obstructive pulmonary disease assessment test, *mMRC* modified Medical Research Council, *FEV1* forced expiratory volume in 1 second, *FVC* forced vital capacity.

*P < 0.05 compared to group I, ^#^*P* < 0.05 compared to group II.

**Fig. 3:**
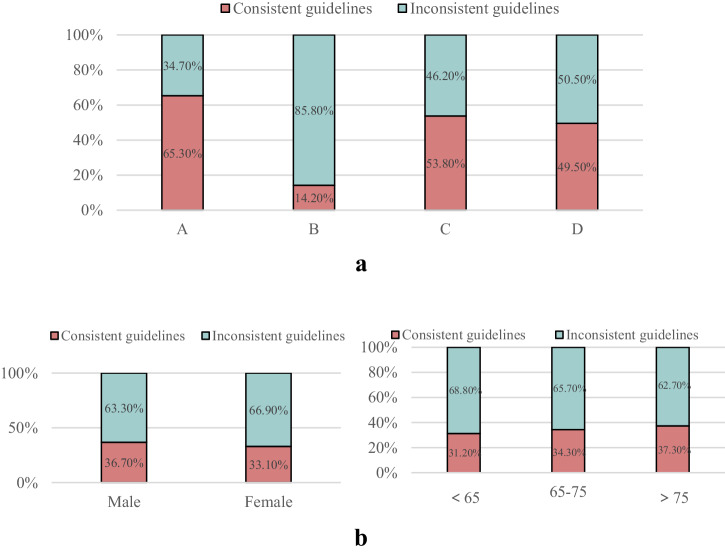
Consistency of initial drug therapy with recommended guidelines. **a** According to the comprehensive assessment grouping criteria, the consistency of the initial drug treatment regimen with the 2017 GOLD guideline recommended regimen. **b** Based on sex and age as grouping criteria, the consistency of the initial drug treatment regimen with the 2017 GOLD guideline recommended regimen.

The compliance rate with guideline recommendations was higher in the GOLD groups A, C and D than in GOLD group B (*χ*^2^ = 140.62, *P* < 0.001) (Fig. 3a). Comparison of adherence to the 2017 GOLD guidelines in patients classified based on sex and age indicated no significant difference in the guideline compliance rate based on sex (*χ*^2^ = 1.041, *P* = 0.308) or age (*χ*^2^ = 1.406, *P* = 0.495) (Fig. 3b).

### Follow-up of the compliance of patients with medication and consistency with the guidelines

**Fig. 4:**
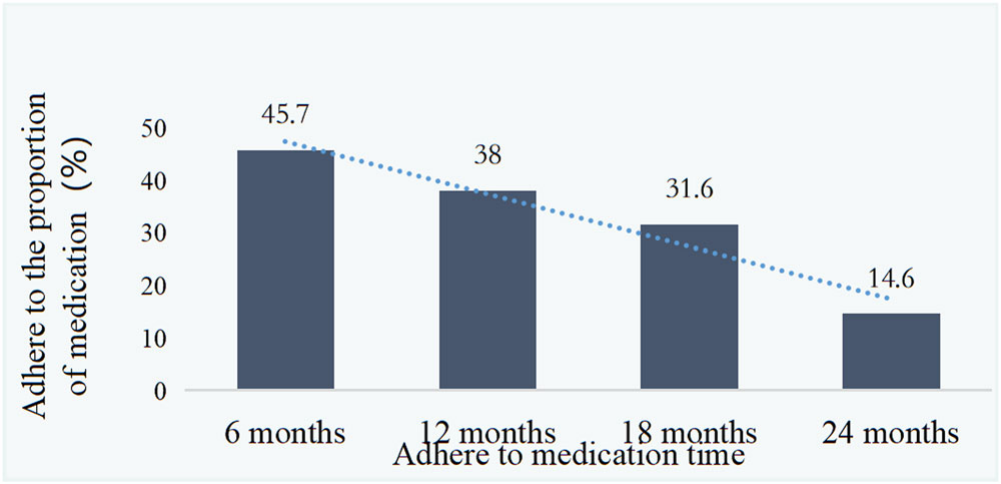
Proportion of patients adhering to medication.

**Fig. 5:**
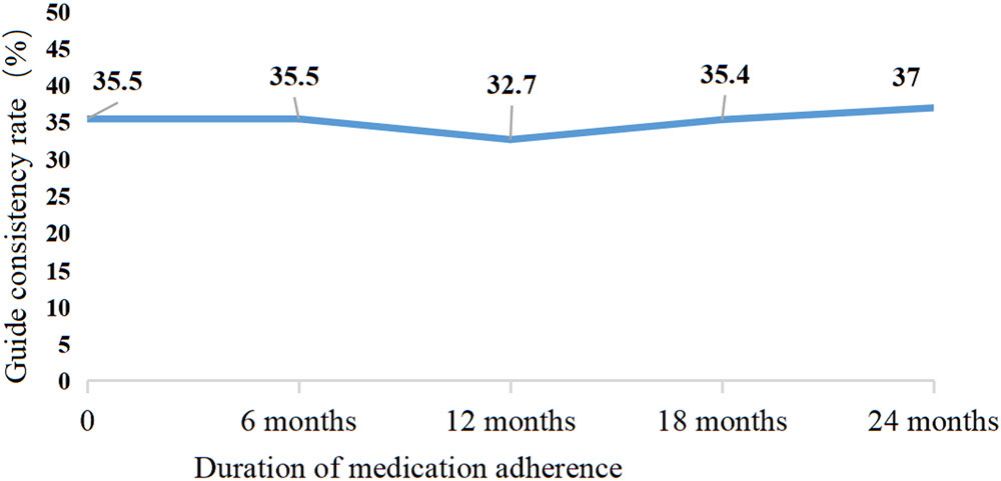
Proportion of patients’ consistency rate with the guideline.

Based on the drug treatment of patients with COPD at the time of enrolment, the proportion of actual drug treatment cases during follow-up was 45.7% after 6 months, 38.0% after 12 months, 31.6% after 18 months and 14.6% after 24 months (Fig. 4). The proportion of patients with medication adherence tended to decrease with the prolongation of follow-up (*P* < 0.05). The consistency of the treatment plan with the guideline was 35.5% (289/814) at the time of enrolment, 35.5% (132/372) at 6 months, 32.7% (101/309) at 12 months, 35.4% (91/257) at 18 months and 37.0% (44/119) at 24 months. However, there was no statistical difference (*χ*^2^ = 1.067, *P* = 0.900) (Fig. 5). The percentage of patients who were under-treated was 49.1% (400/814) at the time of enrolment, 44.8% (167/372) at 6 months, 44.3% (137/309) at 12 months, 43.5% (112/257) at 18 months and 38.6% (46/119) at 24 months. The percentage of patients who were over-treated was 15.4% (125/814) at the time of enrolment, 19.6% (73/372) at 6 months, 22.9% (71/309) at 12 months, 21.0% (54/257) at 18 months and 24.3% (29/119) at 24 months. Both under- and over-treatment are apparent over the course of the follow-up period.

The data presented in Table 3 demonstrate that those with medication adherence for 2 years were disproportionately male and had worse lung function and more hospitalisations compared to the other three groups (*P* < 0.05).

**Table 3:** Baseline characteristics of patients who adhered to treatment regimens during study follow-up.

	6 months	12 months	18 months	24 months	*P* value
	*N* = 372	*N* = 309	*N* = 257	*N* = 119	
Sex					0.047
Male (%)	255 (68.5)*	216 (69.9)*	172 (66.9)*	96 (80.7)	
Female (%)	117 (31.5)	93 (30.1)	85 (33.1)	23 (19.3)	
Age	75.9 ± 8.5	76.5 ± 8.7	76.3 ± 8.8	77.0 ± 9.1	0.486
CAT score	15.96 ± 6.59	15.67 ± 6.69	15.65 ± 6.69	16.67 ± 7.98	0.514
mMRC grade	1.54 ± 0.84	1.52 ± 0.85	1.52 ± 0.85	1.63 ± 0.75	0.520
FEV1%	56.28 ± 29.23*	54.76 ± 31.99	55.50 ± 34.19	47.99 ± 21.28	0.035
FEV1/FVC%	52.15 ± 12.65	50.81 ± 14.66	50.96 ± 14.55	47.01 ± 17.53	0.102
Number of hospitalisations (%)					0.040
<1	214 (57.5)*	196 (63.4)*	156 (60.7)*	58 (48.7)	
≥1	158 (42.5)	113 (36.6)	101 (39.3)	61 (51.3)	
Integrated assessment subgroup (%)					0.427
A	34 (9.1)	30 (9.7)	22 (8.6)	7 (5.9)	
B	176 (47.3)	162 (52.4)	134 (52.1)	50 (42.0)	
C	7 (1.9)	5 (1.6)	4 (1.6)	2 (1.7)	
D	155 (41.7)	112 (36.2)	97 (37.7)	60 (50.4)	

*CAT* chronic obstructive pulmonary disease assessment test, *mMRC* modified Medical Research Council, *FEV1* forced expiratory volume in 1 second, *FVC* forced vital capacity.

* *P* < 0.05 compared to the 24 months group.

We also compared the follow-up data of patients who adhered to treatment regimens for specific time periods. As demonstrated in Table 4, patients who maintained adherence to drug therapy exhibited a range of improvements in their symptoms. The CAT scores of patients who maintained treatment for 6 months improved compared to their score at the time of initial registration to the platform (*P* < 0.05). The proportion of patients in GOLD group A was higher, and the proportion of patients in GOLD group D was lower at 6 months compared to the time of registration to the platform (20.4% vs 9.1% and 33.1% vs 41.7%, respectively; *P* < 0.05 for both). In patients who were on maintenance therapy for 12 months, the CAT score, mMRC grade and number of hospitalisations were improved compared to the initial registration to the platform (*P* < 0.05 for all). Additionally, the proportion of patients in GOLD group A was significantly higher, and the proportion of patients in GOLD group D was significantly lower at 12 months compared to the initial registration to the platform (23.6% vs 9.7% and 23.3% vs 36.2%, respectively; *P* < 0.05 for both). In patients who received maintenance treatment for 18 months, the CAT score and number of hospitalisations were improved compared to the initial registration to the platform (*P* < 0.05 for both). The proportion of patients in GOLD group A was significantly higher, and the proportion of patients in GOLD group D was significantly lower at 18 months compared to the initial registration to the platform (24.5% vs 8.6% and 20.6% vs 37.7%, respectively; *P* < 0.05 for both). In patients who were on maintenance therapy for 24 months, the CAT score, mMRC grade and number of hospitalisations were significantly improved compared to the initial registration to the platform (*P* < 0.05 for all). The proportion of patients in GOLD group A was significantly higher, and the proportion of patients in GOLD group D was significantly lower at 24 months compared to the initial registration to the platform (25.2% vs 5.9% and 21.8% vs 50.4%, respectively; *P* < 0.05 for both).

**Table 4:** Characteristics of follow-up of patients with drug prescriptions.

	Initial	At 6-month follow-up	*P* value	Initial	At 12-month follow-up	*P* value	Initial	At 18-month follow-up	*P* value	Initial	At 24 months follow-up	*P* value
Total cases	*N* = 372	*N* = 372		*N* = 309	*N* = 309		*N* = 257	*N* = 257		*N* = 119	*N* = 119	
CAT score	15.96 + 6.59	14.09 + 5.69	<0.001	15.67 + 6.69	13.95 + 6.9	0.02	15.65 + 6.9	14.05 + 6.38	0.012	16.66 + 7.98	12.66 + 6.40	<0.001
mMRC grade	1.54 + 0.84	1.47 + 0.89	0.220	1.52 + 0.85	1.37 + 0.93	0.018	1.52 + 0.85	1.42 + 0.83	0.126	1.63 + 0.75	1.42 + 0.83	0.036
Number of hospitalisations (%)			0.230			0.001			<0.001			<0.001
<1	214 (57.5)	245 (65.9)		196 (63.4)	233 (75.4)		156 (60.7)	198 (77.0)		58 (48.7)	89 (74.8)	
≥1	158 (42.5)	127 (34.1)		113 (36.6)	76 (24.6)		101 (39.3)	59 (23.0)		61 (51.3)	30 (25.2)	
Integrated assessment subgroup (%)			<0.001			<0.001			<0.001			<0.001
A	34 (9.1)	76 (20.4)*		30 (9.7)	73 (23.6)*		22 (8.6)	63 (24.5)*		7 (5.9)	30 (25.2)*	
B	176 (47.3)	168 (45.2)		162 (52.4)	158 (51.1)		134 (52.1)	133 (51.8)		50 (42.0)	58 (48.7)	
C	7(1.9)	5 (1.3)		5 (1.6)	6 (1.9)		4 (1.6)	8 (3.1)		2 (1.7)	5 (4.2)	
D	155 (41.7)	123 (33.1)*		112 (36.2)	72 (23.3)*		97 (37.7)	53 (20.6)*		60 (50.4)	26 (21.8)*	
Guideline compliance rate (%)			0.277			0.798			0.854			0.290
Consistent	118 (31.7)	132 (35.5)		104 (33.7)	101 (32.7)		93 (36.2)	91 (35.4)		52 (43.7)	44 (37.0)	
Non-compliance	254 (68.3)	240 (64.5)		205 (66.3)	208 (67.3)		164 (63.8)	166 (64.6)		67 (56.3)	75 (63.0)	

*CAT* chronic obstructive pulmonary disease assessment test, *mMRC* modified Medical Research Council.

*P < 0.05 compared to initial entry to the platformwith drug prescriptions.

### Follow-up of patients with initial medication regimens consistent with the 2017 GOLD guidelines

In the initial cohort of 289 patients who consistently followed the guidelines, 40.8% (118/289) of the patients adhered to drug therapy after 6 months and 66.1% (78/118) of the patients maintained guideline adherence. After 12 months, 36.0% (104/289) of the patients adhered to drug therapy and 52.9% (55/104) of these patients maintained guideline adherence. After 18 months, 32.2% (93/289) of patients adhered to drug therapy and 47.3% (44/93) of the patients maintained guideline adherence. After 24 months, 18.0% (52 out of 289) of the patients adhered to drug therapy and 46.2% (24/52) of the patients maintained guideline adherence. Thus, the adherence rate to drug therapy in patients whose initial treatment adhered to guidelines decreased during the follow-up period (*P* < 0.05).

As shown in Table 5, patients who maintained adherence to medical therapy experienced a considerable improvement in symptoms during the follow-up period. Analysis of the follow-up data revealed that the patients who adhered to treatment for 6 months had better CAT scores and fewer hospitalisations compared to the initial registration to the platform (*P* < 0.05 for both). The patients who adhered to treatment for 12 months had significantly fewer hospitalisations compared with the initial registration to the platform (*P* < 0.05). Additionally, the proportion of patients in GOLD group B was higher, and the proportion of patients in GOLD group D was lower at 12 months compared to the initial registration to the platform (44.2% vs 26.0% and 27.9% vs 52.9%, respectively; *P* < 0.05 for both). Similarly, the patients who adhered to treatment for 18 months had significantly fewer hospitalisations compared to the initial registration to the platform (*P* < 0.05). Furthermore, the proportion of patients in GOLD group B was significantly higher, and the proportion of patients in GOLD group D was significantly lower at 18 months compared to the initial registration to the platform (46.2% vs 30.1% and 29.0% vs 53.8%, respectively; *P* < 0.05 for both). Finally, the patients who adhered to treatment for 12 months exhibited significant improvement in CAT scores and mMRC grades and had significantly fewer hospitalisations (*P* < 0.05 for all). The proportions of patients in GOLD groups A and B were significantly higher, and the proportion of patients in GOLD group D was significantly lower compared to the initial registration to the platform (26.9% vs 5.8%, 42.3% vs 23.1% and 26.9% vs 69.2%, respectively; *P* < 0.05 for all).

**Table 5:** Comparison of follow-up data of patients treated with inital medications consistent with the 2017 GOLD guidelines.

	Initial	At 6-month follow-up	*p* value	Initial	At 12 months follow-up	*P* value	Initial	At 18 months follow-up	*P* value	Initial	At 24 months follow-up	*P* value
Total cases	*N* = 118	*N* = 118		*N* = 104	*N* = 104		*N* = 93	*N* = 93		*N* = 52	*N* = 52	
CAT score	16.08 ± 7.66	14.15 ± 6.92	0.043	15.57 ± 7.67	14.08 ± 6.44	0.130	16.15 ± 7.57	14.98 ± 6.86	0.270	17.77 ± 8.39	13.35 ± 7.04	0.004
mMRC grade	1.52 ± 0.83	1.57 ± 0.88	0.650	1.57 ± 0.87	1.46 ± 0.91	0.393	1.61 ± 0.82	1.45 ± 0.89	0.201	1.75 ± 0.68	1.38 ± 0.87	0.019
Number of hospitalisations (%)			0.037			0.001			0.001			O.001
<1	47 (39.8)	63 (53.4)		48 (46.2)	71 (68.3)		41 (44.1)	63 (67.7)		16 (30.8)	36 (69.2)	
>1	71 (60.2)	55 (46.6)		56 (53.8)	33 (31.7)		52 (55.9)	30 (32.3)		36 (69.2)	16 (30.8)	
Integrated assessment subgroup (%)			0.116			0.001			0.006			O.001
A	22 (18.6)	29 (24.6)		19 (18.3)	25 (24.0)		13 (14.0)	20 (21.5)		3 (5.8)	14 (26.9)*	
B	23 (19.5)	34 (28.8)		27 (26.0)	46 (44.2)*		28 (30.1)	43 (46.2)*		12 (23.1)	22 (42.3)*	
C	4 (3.4)	4 (3.4)		3 (2.9)	4 (3.8)		2 (2.2)	3 (3.2)		1 (1.9)	2 (3.8)	
D	69 (58.5)	51 (43.2)		55 (52.9)	29 (27.9)*		50 (53.8)	27 (29.0)*		36 (69.2)	14 (26.9)*	

*CAT* chronic obstructive pulmonary disease assessment test, *mMRC* modified Medical Research Council.

*P < 0.05 compared to initial registration to the platform.

## Discussion

The GOLD Global COPD Diagnosis and Treatment Strategy Report recommends that all patients should take at least one drug during the stable phase of COPD to obtain symptomatic relief and reduce the incidence of adverse outcomes^16^. The standardised use of inhaled drugs is the first choice of treatment among patients with COPD, as these drugs can considerably improve clinical symptoms and prognosis in patients with moderate/severe COPD^17^. By contrast, insufficient treatment heightens the likelihood of acute exacerbations^18^. Therefore, if conditions permit, inhalation therapy can be more effective than oral therapy; however, this has not been observed in the actual clinical setting. More than one-third of patients with COPD in Spain do not adhere to the 2017 GOLD guideline recommendations^7^. A Swiss study on the implementation of GOLD by GPs in community healthcare settings showed that 44% of patients with COPD did not comply with the GOLD-recommended treatment regimen, and the compliance rate of GPs to implement GOLD was low^19^. A retrospective study of patients with COPD from northern Italy reported that only 38.7% of patients were receiving treatment^20^. Another study showed that only about 30% of patients with COPD were treated in accordance with the guidelines. According to the comprehensive assessment, the consistency with the guidelines was 20.6% in group A, 32.3% in group B, 5.9% in group C and 39.2% in group D^21^. Similar findings were reported in studies from other countries^22,23^.

Only a few studies have focused on the pharmacotherapy of patients with COPD in China. Liang et al.^24^ reported that less than 20% of patients with COPD in China adhered to the drug therapy after discharge, and Wang et al.^12^ indicated that the consistency between drug therapy and guidelines was less than 3.5%. There has been a slight increase in the number of reports on the pharmacotherapy of patients with COPD in recent years. Chen et al.^13^ reported that 10.8% of patients with COPD were highly adherent to the drug therapy (PDC ≥ 0.8) during the 12-month follow-up management. Another study focused on medication adherence among patients with COPD in China found that only 33.2% of patients adhered to medication at 1-year follow-up^25^. A recent COPD study showed that 27.7–35.4% of patients in tertiary hospitals and 28.9–36.4% of patients in secondary hospitals accepted ICS/LABA; further, 18.3–19.5% and 22.1–24.6% patients in tertiary hospitals and 12.5–12.6 and 12.2–13.7% patients in secondary hospitals received LAMA monotherapy and ICS/LABA + LAMA, respectively. Moreover, the proportion of patients receiving the above treatment regimens was almost the same at 1-year follow-up^14^. At present, limited literature is available regarding the management of COPD by general practitioners.

This study collected real data on COPD management by GPs through MICCOPD. Out of the total 2044 patients diagnosed with COPD, only 814 individuals (39.8%) were prescribed an initial medication regimen. During the 2 consecutive years of 814 patients follow-up management, the drug treatment compliance rates of patients were 45.7%, 38%, 31.6% and 14.6% at 6, 12, 18 and 24 months, respectively. Patients adherence to drug treatment tended to decline annually. Despite initial adherence to drug regimens consistent with the guidelines, patient adherence to drug therapy declined during subsequent follow-up visits. According to the guidelines for the prevention and treatment of COPD by the China Respiratory Community Alliance^26^, patients with newly diagnosed COPD must be diagnosed again by specialists, and the diagnosis and treatment plan should be formulated before they can be prescribed in the community, which affects the diagnosis and treatment of patients who cannot visit the specialist’s clinic in time. Furthermore, a considerable proportion of patients with COPD lacked awareness of the seriousness of their condition and had limited knowledge regarding preventative measures and treatment options^27^. This lack of knowledge may lead to premature discontinuation of prescribed medications, particularly among individuals experiencing mild dyspnea symptoms in stable environments. These patients may be reluctant to seek further care from respiratory specialists. In addition, some patients may decline participation in specialized treatment programs owing to concerns about reliance on inhaled medications. Our study findings indicate that patients who adhered to medication were typically older, with frequent hospitalizations and severe illness.

The results showed that 35.9% and 41.9% of patients inhaled bronchodilators and took oral bronchodilators as the initial drug treatment, respectively. The overall initial treatment compliance with the 2017 GOLD guidelines was only 35.5% in this study, whereas 49.1% and 15.4% of the patients were under- and over-treated, respectively. A considerable proportion of COPD patients in groups A and B were prescribed ICS + LABA inhalers, which was a deviation from the GOLD2017 guidelines and indicative of overtreatment. Conversely, the initiation of recommended inhalation therapy was delayed in patients in groups C and D, who opted for intermittent oral medications instead, leading to inadequate treatment outcomes. Over- and under-treatment are also common clinical problems. In a Swiss study, 53% of the patients were not taking medications consistent with the 2017 GOLD recommendations. In that study, 87.1%, 37% and 47.5% of the patients in GOLD groups A, B and C, respectively, were over-treated, whereas 15.2% of the patients in GOLD group D were not receiving adequate treatment according to the 2017 GOLD guidelines^6^. A study of patients with COPD in Canada who did not adhere to recommended treatment regimens revealed a prevalent lack of implementation^28^. Factors contributing to the non-adherence in low-risk patients included female gender, high socioeconomic status, long COPD duration, increased comorbidities, presence of dementia, history of mental health conditions and old physician age. Factors contributing to non-adherence in high-risk patients included advanced age, multiple comorbidities, cognitive impairment, heart failure, psychiatric disorders and the age of the treating physician. However, individuals at high risk were inclined to adhere to prescribed medications, possibly owing to a shared goal between healthcare providers and patients to effectively manage the condition and mitigate disease severity.

In patients who adhered to medication, the percentage of patients whose medication aligned with the recommended treatment plan was 35.5% at 0.5 years, 32.7% at 1 year, 35.4% at 1.5 years and 37.0% at 2 years. The overall proportion of patients who adhered to the recommended treatment plan was relatively stable. Under- and over-treatment also persisted during the follow-up period, with no considerable improvement. The persistent lack of adherence to the guidelines may be owing to the lack of community practitioner adherence to the guidelines. General practitioners may not promptly modify drug regimens based on patient symptoms, particularly in elderly patients with COPD with comorbidities, which may diminish patient survival rates. Analysis of a survey administered to general practitioners in primary care settings in Sweden and Canada revealed notable deficiencies in understanding multiple facets of COPD diagnosis and treatment and substandard adherence to the 2017 GOLD guidelines^29,30^. In addition, we previously demonstrated that knowledge about COPD prevention and management among general practitioners was insufficient^31^. Various factors impact medication adherence in patients with COPD, highlighting the importance of improving general practitioner training and adherence to COPD guidelines, enhancing professional competence and fostering better communication between patients and healthcare providers, including doctors and pharmacists.

COPD is a chronic disease, and long-term adherence to standardised drug therapy is crucial for its prevention and treatment. Every patient with COPD hopes to control their symptoms as soon as possible and reduce the number of acute exacerbations in the future. This study showed that adherence to drug therapy for a long duration could improves CAT scores, mMRC classification grade and the number of hospitalisations. During 2 years of follow-up, the proportion of patients in group D tended to decrease compared with the initial data. Various domestic and foreign studies have shown that the compliance rate of patients with COPD with drug therapy is 40%–60%, and the consistency between the actual drug therapy and guidelines fluctuates between 25% and 48%. The results of our study suggested that the consistency of medication adherence with the guidelines in Shanghai Songjiang was better than those reported in previous studies conducted in China^12^. Nevertheless, several obstacles to the effective management of COPD during the stable phase remain. Treatment adherence and alignment of drug treatment regimens with guidelines have not been optimized and lag behind the COPD management practices in developed countries. The prevention and treatment capabilities of general practitioners should be enhanced. In addition, we propose adjustments to the basic guidelines to grant qualified general practitioners the authority to prescribe initial treatment for COPD. This measure could expedite drug treatment for a greater number of patients. The combination of general practitioner–specialist doctor–patient prevention and treatment should be strengthened pharmacists, nurses and prevention healthcare professionals should cooperate to improve the compliance with standardised drug treatment in patients with COPD.

Our study has several limitations. First, this is a retrospective cohort study. Second, as all patients were from Shanghai, they cannot represent the general situation in China. Third, the study could not capture the fluctuating changes in pulmonary function throughout the follow-up period owing to limitations related to community-based pulmonary function testing. Finally, due to the outbreak of the coronavirus disease 2019 pandemic in 2020, this study only included data collected during the 2-year follow-up after registration to the platform. Hence, in future studies, the MICCOPD platform will be used to collect pulmonary function data and health economics data from follow-up patients to establish a solid groundwork for improving patient treatment adherence and standardised care aligning with the guidelines. However, challenges in COPD management during stable periods persist. Consequently, further research is warranted to explore and address these challenges.

In summary, based on the MICCOPD, the compliance and guideline consistency rates among patients with COPD in the community who were managed by GPs are higher than those previously reported in China; however, there is still a big gap compared with developed countries. Compliance was more common in male patients, patients with poor lung function and patients with more emergency department admissions. The number of acute exacerbations, decrease in comprehensive assessment grade, and symptoms in patients with COPD can be improved by ensuring adherence to the drug treatment. Enhancing general practitioner proficiency in the prevention and management of COPD and increasing patient awareness of the condition are crucial for standardising and improving adherence to initial and follow-up COPD treatments.

## Methods

### Ethical considerations

The present study was approved by the Ethics Committee of Shanghai Songjiang District Central Hospital in accordance with the ethical requirements of the Declaration of Helsinki (China Clinical Trials Registry registration number: ChiCTR2000031092). The confidentiality of the personal information of all participants was preserved. Signed informed consent was not required due to the retrospective and observational study design.

### Study design

This is a retrospective cohort study including data on COPD management obtained from the regional COPD Information Centre platform in Songjiang District. This platform, a mobile internet management system for patients with COPD, involves GPs from 21 community health service centres and 21 respiratory specialists from 3 general hospitals in Songjiang District^32^. Background information of patients recorded in the COPD management information centre included the following: (1) basic information on health records (name, sex, age, home address, contact phone number, ID number, health insurance number, lung function at enrolment, lung function classification and COPD assessment test [CAT] score), modified Medical Research Council (mMRC) grade; (2) COPD medication information (drug name, dosage, prescription time and medication course); (3) follow-up information obtained by the family physician, including lung function (forced expiratory volume in 1 second [FEV1], forced vital capacity [FVC], FEV1/FVC, FEV1/projected value), lung function grade, CAT score, mMRC grade and hospitalisation registry, among others. Higher CAT scores and mMRC grades indicated more severe COPD symptoms and dyspnoea, respectively.

#### Data acquisition and inclusion and exclusion criteria

Inclusion criteria were as follows: (1) lung function tests meeting the 2017 GOLD diagnostic criteria for COPD^16^, in which the COPD diagnosis was based on the presence of a post-bronchodilator FEV1/FVC of <0.70 in patients with appropriate symptoms following significant exposure to noxious stimuli; (2) availability of complete medication records at the time of registration to the platform between 1 January 2017 and 31 December 2020.

Exclusion criteria were as follows: (1) no medication regimen or incomplete information on medication use at the time of registration to the platform and (2) follow-up evaluations not performed at predefined times or missing assessment indicators in follow-up records.

#### Follow-up of community GPs on COPD

In the present study, patients were categorised into four groups. Patients in GOLD groups A and B were evaluated once every six months, and those in GOLD groups C and D were evaluated once every three months. After discharge, patients who sought care at the emergency department or were admitted to the hospital for an acute exacerbation of COPD were monitored for 1 month, with subsequent follow-up intervals based on illness severity. During each follow-up visit, the primary care physician evaluated the status of patients with COPD, including administering the COPD Assessment Test (CAT), determining the modified Medical Research Council (mMRC) scores, documenting the frequency of emergency hospitalizations within the previous year, and reclassifying the severity of COPD based on the GOLD 2017 comprehensive assessment criteria.

#### Pharmacotherapeutic approaches of MICCOPD

Drug treatment recommendations based on the 2017 GOLD criteria were followed. Specifically, the preferred treatment in GOLD group A included bronchodilators, and efficacy evaluation was performed to determine whether to continue, stop or replace the medication. Preferred treatment in GOLD group B included long-acting bronchodilators, either long-acting beta-agonists (LABAs) or long-acting muscarinic antagonists (LAMAs); combination therapy with LAMAs plus LABAs could be used for persistent symptoms. Preferred treatment in GOLD group C included LAMAs as the first choice; in patients with persistent or worsening symptoms, LAMAs plus LABAs or LABAs plus ICS could be used. Preferred treatment in GOLD group D included LABAs plus LAMAs or LABAs plus ICS; in patients with continually worsening symptoms, LABAs in combination with LAMAs and ICS could be used. Additionally, roflumilast or macrolide antibiotics could be added in patients with continually worsening symptoms. Compliance with the 2017 GOLD guideline recommendations was determined by comparing the actual medication regimens with these recommendations.

#### Definitions of under- and over-treatment per the guidelines

According to the patient’s drug treatment plan^16^, the definitions were consistent with the first and second recommended drug treatment plans of GOLD 2017 COPD.

Over-treatment was defined as the administration of a different type of drug or a higher dose of the actual therapeutic drug than the recommended dose per the guidelines (e.g., when a patient assessed suitable for Group B received the drug treatment administered to patients in Group D).

Under-treatment was defined as the administration of a different type of drug or lower dose of the actual therapeutic drugs than the recommended one per the guidelines (e.g., when a patient was assessed suitable for Group D received the drug treatment administered to patients in Group B).

#### Statistical analysis

All statistical analyses were conducted using SPSS (version 24; IBM, Armonk, NY, USA). All baseline characteristics were presented as descriptive data. In the overall study cohort, data were presented as means with standard deviation or numbers and percentages. Normally distributed data were compared using Student’s *t* test or one-way analysis of variance, and non-normally distributed data were compared using non-parametric tests such as Kruskal–Wallis *H* test or Mann–Whitney *U* test. Categorical data were compared using the chi-square test. A *P* value of <0.05 was considered to indicate statistical significance.

### Supplementary information

nr-reporting-summary

## Data Availability

The datasets used and/or analysed during this study are available from the corresponding author upon reasonable request.
